# Role of miRNA *Let-7* and Its Major Targets in Prostate Cancer

**DOI:** 10.1155/2014/376326

**Published:** 2014-09-03

**Authors:** Siegfried Wagner, Anaclet Ngezahayo, Hugo Murua Escobar, Ingo Nolte

**Affiliations:** ^1^Small Animal Clinic, University of Veterinary Medicine Hannover, 30559 Hannover, Germany; ^2^Institute of Biophysics, University Hannover, 30419 Hannover, Germany; ^3^Division of Medicine, Department of Haematology/Oncology, University of Rostock, 18057 Rostock, Germany

## Abstract

Prostate cancer is worldwide the sixth leading cause of cancer related death in men thus early detection and successful treatment are still of major interest. The commonly performed screening of the prostate-specific antigen (PSA) is controversially discussed, as in many patients the prostate-specific antigen levels are chronically elevated in the absence of cancer. Due to the unsatisfying efficiency of available prostate cancer screening markers and the current treatment outcome of the aggressive hormone refractory prostate cancer, the evaluation of novel molecular markers and targets is considered an issue of high importance. MicroRNAs are relatively stable in body fluids orchestrating simultaneously the expression of many genes. These molecules are currently discussed to bear a greater diagnostic potential than protein-coding genes, being additionally promising therapeutic drugs and/or targets. Herein we review the potential impact of the microRNA *let-7* family on prostate cancer and show how deregulation of several of its target genes could influence the cellular equilibrium in the prostate gland, promoting cancer development as they do in a variety of other human malignant neoplasias.

## 1. Introduction

Prostate cancer (PC) is a heterogeneous disease ranging from an asymptomatic to a fatal systemic malignancy [1]. According to the World Health Organization (WHO) 1,111,689 men were estimated to be diagnosed with PC in the year 2012 (http://globocan.iarc.fr/). Accounting worldwide for 6.6% (307,471) of all cancer death in men in 2012, PC is one of the most common malignant neoplasias and the sixth leading cause of cancer related death in men (http://globocan.iarc.fr/).

The development of PC is considered to be a multistep process initiated by genetic and epigenetic changes [1]. Human PC is commonly accepted to be an androgen dependent malignancy.

An analysis of PC related metastatic pattern in 1,589 patients by Bubendorf et al. revealed that 35% of the analyzed tumors spread to other organs with preference to the bones (90%), lungs (46%), liver (25%), pleura (21%), and adrenals (13%) [2].

The androgen deprivation therapy is actually the most effective palliative standard treatment for primary advanced PCs with bone metastasis (effective in up to ~90% of patients). However, the great majority of patients relapse subsequently due to the development of castration resistance [3].

Since the introduction of the prostate-specific antigen (PSA) test in the 1990s, the number of diagnosed cases has been rapidly rising being initially associated with a reduced mortality. However, the recent decline in PC related mortality rates is now being discussed to be partially explained by the improved treatment and earlier diagnosis due to a broad standard PSA screening in economically developed countries [4, 5]. As the standard PSA screening in the early diagnosis of human PC remains a very controversial issue, novel, reliable molecular PC markers are needed [6–8].

A promising marker candidate gene is the miRNA* let-7*, which was reported to be down regulated among others in human PC [9–11]. Further, the reconstitution of the* let-7* expression resulted in suppression of PC cell proliferation [10, 12]. In general a single miRNA is able to regulate a huge number of genes. Concerning* let-7* the respective acting ways are actually not entirely deciphered.

Nevertheless, it is to be expected that a deeper understanding of the molecular interactions of* let-7* and associated genes will significantly contribute to the development of novel diagnostic and therapeutic treatment modalities for PC.

Due to the complex regulation mechanisms of* let-7* and its potential role in PC development and relapse the present review highlights* let-7* and its direct and downstream targets in the context of PC.

## 2. Micro RNA* Let-7* Family

**Figure 1:**
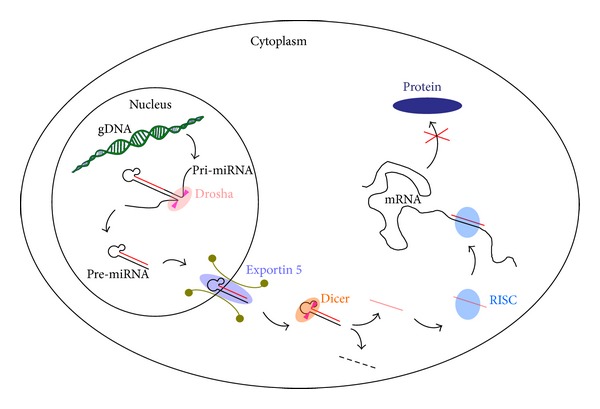
Schematic overview of the miRNA maturation and the way of function in eukaryotic cells. The endogenously expressed primary miRNA (pri-miRNA) is processed in the nucleus of a cell by the enzyme Drosha. The emerging precursor miRNA (pre-miRNA) product is exported into the cytoplasm by Exportin-5 and maturated by Dicer. Finally the guide strand is incorporated into the RNA-induced silencing complex (RISC), which blocks the translation of the target mRNA.

MicroRNAs (miRNAs) are small, non-protein-coding RNAs derived from long, endogenously expressed primary RNA (pri-miRNA) molecules. These pri-miRNAs are processed by the nuclear enzyme Drosha to precursor RNAs (pre-miRNAs), exported by Exprotin-5 [13] and maturated by the cytoplasmic enzyme Dicer [14]. Finally the guide strand of the mature miRNA is incorporated into the RNA-induced silencing complex (RISC), which blocks the translation of the target mRNA by binding to its 5′-, 3′-prime, or exon regions [15, 16]. The passenger strand is usually degraded [17] (Figure 1).

Mature miRNAs are known to be part of the gene expression regulating machinery at transcriptional [18, 19] and as well posttranscriptional level [13]. It was reported that a single miRNA can orchestrate the expression of several genes and a single gene can be regulated by a set of different miRNAs [20–22]. Several observations suggest that more than 60% of all protein coding genes are regulated by miRNAs [23].

One of the first described members of the large class of non-protein-coding RNAs is* let-7* which was the second miRNA discovered and designated as* lethal-7* (*let-7*) according to the phenotype of a* let-7* deficient* C. elegans* mutant [20]. Soon thereafter, further* let-7* homologs were identified in a variety of species ranging from vertebrates to mollusks [24].

In contrast to “less complex” organisms such as worms, vertebrates show a higher number of* let-7* isoforms coded by different genes [16]. In humans, 13* let-7* family precursor miRNAs were described (*let-7a-1*,* let-7a-2*,* let-7a-3*,* let-7b*,* let-7c*,* let-7d*,* let-7e*,* let-7e*,* let-7f*,* let-7g*,* let-7i*,* miR-98,* and* mir-202*) which code for 10 different mature* let-7* miRNA isoforms [25].

Although the role of* let-7* is still not fully understood, it is evident that the* let-7* family members have a distinct expression pattern in animal development [26]. In the embryonic stage the* let-7* miRNAs were found to be barely detectable, but having an increased expression in differentiated cells [20, 27]. Furthermore, aberrant* let-7* expression was associated with a variety of human diseases as, for example, cardiovascular diseases [28], liver fibrosis [29], lung diseases [30], and cancer [9–12, 26, 31–34]. Interestingly several* let-7* family members were found to be located at fragile sites of human chromosomes potentially contributing to aberrant* let-7* transcript levels [35].

Cancer initiation, progression, and aggressiveness are hypothesized to be driven by cancer stem cells (CSCs) [36, 37]. Inflammatory microenvironment [38] as well as epithelial-to-mesenchymal transition (EMT), which is tightly linked with CSC biology [39], seems to play a substantial role in cancer etiology as well. Remarkably, a linkage between these factors is the* let-7* miRNA family. As described above* let-7* was shown to be downregulated in prostatic CSCs [36] whereas reconstitution of the* let-7* suppressed the growth of PC cells [10, 12]. Additionally, a direct causal link between cancer and inflammation is given by the association of* let-7*, IL6, and NF*κ*B, which are major players involved in the epigenetic switch from inflammation to cell transformation [31]. The connection between EMT and* let-7* is represented by the* HMGA1 *and* HMGA2 *genes, which are directly regulated by* let-7* and were found to be implicated in EMT [40, 41].

Further, miRNAs of the* let-7* family were reported to directly, negatively regulate* IL6 *[24],* NRAS* [42],* c-Myc, HMGA1 *[43, 44],* HMGA2 *[45], and* CCND2 *[11]. Notably, these* let-7* targets are involved in a wide range of diverse cellular processes interwoven with* let-7* and each other in a fine balanced way (Figure 10).

The c-Myc protein regulates the biogenesis of* let-7 *by stimulating* Lin28 *[46], Lin28 in turn blocks the maturation of* let-7 *[47]. Additionally, c-Myc stimulates the expression of* HMGA1 *[48],* AR *[12], and* IL6* [49]. NRAS is suggested to have an impact on* HMGA2 *biogenesis [45].* HMGA2* on the other hand influences* HMGA1,* its gene product in turn regulates the expression of* c-Myc *[50] and* HMGB1 *[51]. HMGB1 was found to bind the* AR* promoter [52], AR protein was described itself to stimulate* let-7* expression [53] (Figure 10).

Interestingly, the* let-7 *family [10, 11] and some of its above mentioned targets were already found to be implicated in PC. As* let-7* is linked with all these protein-coding genes a deeper insight into these connections is of great interest. Thus, these interactions are reviewed more detailed in the following parts.

## 3. *HMGA1*

The high mobility group proteins (HMG) are chromatin associated nonhistone proteins constituting three superfamilies (HMGA, HMGB, and HMGN) which are classified by their characteristic functional DNA-binding motifs [54]. Expression of these proteins was described to be involved in a variety of biological processes as, for example, transcription, embryogenesis, differentiation, neoplastic transformation, apoptosis, and inflammation [52, 55, 56].

**Figure 2:**
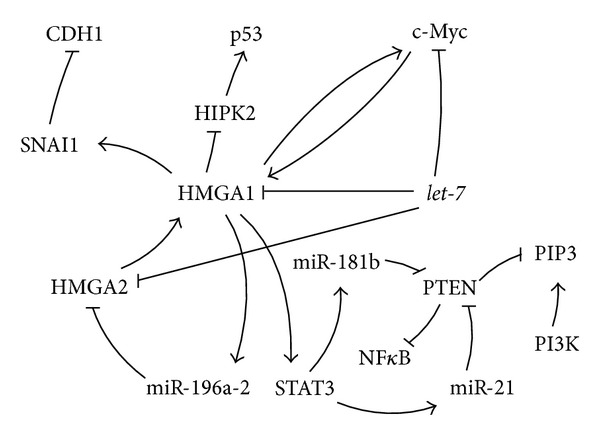
Overview of the described interactions between* let-7* and its direct target* HMGA1 *with other genes.

In human neoplasias the HMGA genes are among the most commonly rearranged genes [57]. Deregulation of the* HMGA1* expression was described in human PC [58, 59], lung cancer [60], and breast cancer cells [61]. Its oncogenic property is speculated to be partly mediated through the cytoplasmic relocalization of the HIPK2 which is a proapoptotic activator of the tumor suppressor p53 [51] (Figures 2 and 10). HMGA1 was reported to enhance the proliferation rate and invasion of PC cells [62, 63] potentially through the implication in epithelial-to-mesenchymal transition (EMT) [61]. In line with this,* HMGA1* knock down in the human triple-negative breast cancer cell lines MDA-MB-231 and Hs578T repressed the mesenchymal gene* SNAI1* and stimulated* CDH1* expression [40] (Figures 2 and 10), both of which are involved in EMT [64, 65]. Furthermore, HMGA1 was reported to drive tumor progression by reprogramming cells to a stem-cell-like state [40]. In accordance, Shah et al. reported in human embryonic stem cells (hESCs) a significant downregulation of the stemness-associated genes* OCT4*,* Sox2*,* Lin28,* and* c-Myc* 96 h after* HMGA1* repression [50]. Interestingly HMGA1 is not only stimulating* c-Myc* expression [50] it was also reported to be itself induced by c-Myc [48] (Figures 2 and 10). It is remarkable that HMGA1 is implicated in the upregulation of several miRNAs in murine embryonic fibroblasts. Among these miRNAs is the* miR-196a-2,* which in turn is predicted to target its sister gene* HMGA2* [66] (Figures 2 and 10). Furthermore, Hillion and colleagues reported a positive correlation between HMGA1 and STAT3 in a subset of primary human acute lymphoblastic leukemia samples [67]. In line with this, HMGA1 was described to bind the STAT3 promoter and to upregulate its expression in malignant human hematopoietic cells [67] (Figures 2 and 10). The transcription factor STAT3 mediates uncontrolled growth, angiogenesis, and survival of cells and has a great potential as target in cancer therapies [68]. Remarkably, Iliopoulos et al. identified STAT3 binding sites in the promoters of the miRNAs* miR-181b* and* miR-21 *[69] (Figures 2 and 10). These tiny regulators in turn where found to block* PTEN* (Figures 2 and 10), stimulating the activity of NF*κ*B [69]. The tumor suppressor PTEN functions as an antagonist of PI3K by dephosphorylating its product PIP3 [70] (Figure 10).

The* HMGA1* and* HMGA2* genes were reported to be highly expressed during embryogenesis, reexpressed in several cancer types but to be absent or not detectible in most of the adult healthy tissues [57, 71]. The expression of both* HMGA1*,* HMGA2,* and of its regulator* let-7* was shown to be negatively correlating in gastroenteropancreatic neuroendocrine tumors [44] and retinoblastomas [72]. In accordance they were found to be directly, negatively regulated by* let-7* [45, 73, 74] (Figures 2 and 10).

## 4. *HMGA2*

**Figure 3:**
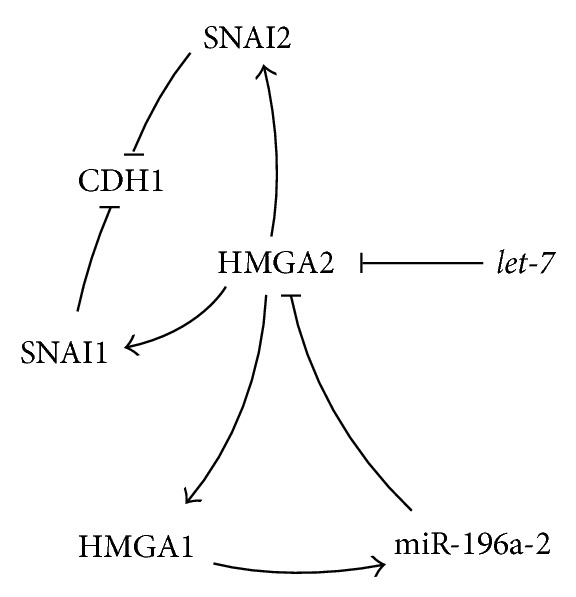
Association of the HMGA2/*let-7* axis with the regulation of genes involved in EMT and miRNAs.

Comparable to the described* HMGA1* knock down, the repression of* HMGA2* in the human PC cell line PC-3 induced an upregulation of* CDH1* indicating an important role in EMT [41].* SNAI1* and* SNAI2* are repressors of* CDH1* and were shown to be directly activated by HMGA2 [45] (Figures 3 and 10).

Similar to* HMGA1*, an upregulation of* HMGA2* was reported in human lung and breast cancers [75, 76] as well as in a subset of canine PCs [77]. Furthermore,* HMGA2* was recently described to modify gene expression not only as protein but as well as a competing endogenous RNA (ceRNA) by acting as a decoy for mature* let-7* miRNAs [78]. Interestingly, a stimulating HMGA2 influence on the expression of its sister protein HMGA1 was found in rat epithelial thyroid cells [79], thus constituting a feedback loop by the stimulation of its suppressor, the* miRNA-196a-2* [66] (Figures 3 and 10). Remarkably* HMGA2* was described to bear seven* let-7*-binding sites in its 3′-untranslated region (3′-UTR) [33]. Aberrations of the chromosomal region 12q14-15 that affect* HMGA2* were frequently found in human cancers [80–82]. Moreover, the disrupted pairing between* let-7* and* HMGA2* by mRNA truncations of the 3′UTR was reported to induce HMGA2 overexpression leading to tumor formation [33].

## 5. *HMGB1*

The high mobility group box 1 (HMGB1) is one of the HMGB superfamily members which was also shown to be implicated in inflammation exercising cytokine like functions [83]. In line with its multiple roles it can be located in the nucleus as well as in the cytoplasm and can even be released passively by necrotic cells or actively secreted in response to inflammatory signals by certain cell types [83, 84].

**Figure 4:**
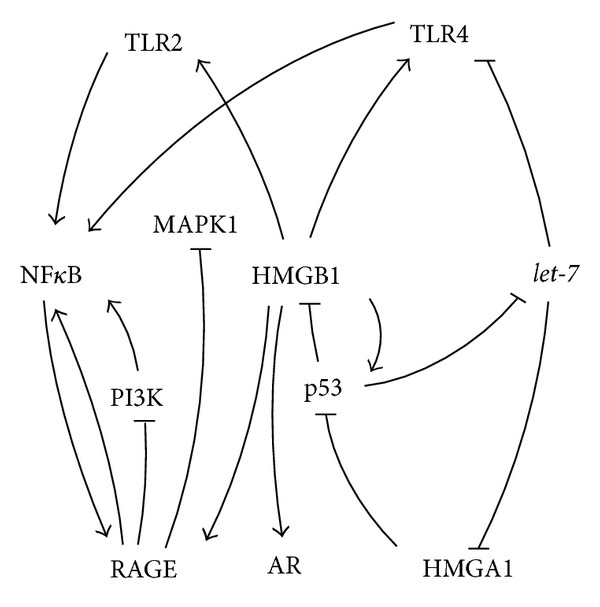
Schematic overview over the HMGB1 and* let-7* regulatory pathways affecting each other's activity.

This proinflammatory cytokine exerts its function by interacting with the toll-like receptors (TLR) 2, and TLR4 and RAGE [85–87] (Figures 4 and 10). Interestingly, the receptor coding gene* TLR4* was found to be a direct* let-7i* target (Figure 4), presenting a mechanism to modify the HMGB1 signaling [88]. The activation of the HMGB1 receptor RAGE results among others in deactivation of MAPK1 and PI3K [89]. PI3K in turn was shown to stimulate NF*κ*B [90]. Furthermore, the TLRs and RAGE were demonstrated to activate NF*κ*B thus, inducing the secretion of angiogenic factors, growth factors, and cytokines [85, 91].

Remarkably, NF*κ*B is able to stimulate RAGE expression by binding to its promoter constituting a positive feedback loop [92] (Figures 4 and 10). Blockade of the RAGE/HMGB1 signaling decreased growth and metastasis of implanted and as well of spontaneously developing tumors in susceptible mice [93].

HMGB1 was described to be involved in all proposed hallmarks of cancer and is thus a potential target for therapeutic and diagnostic approaches [94]. Kuniyasu et al. observed the secretion of HMGB1 in primary cultured human prostatic stromal cells after androgen deprivation [95].* In vitro* suppression of HMGB1 was demonstrated to block the invasion of PC-3 cells which was reversed by culturing the cells in conditioned medium of the above-mentioned stromal cells deprived of androgen [95, 96]. Additionally, HMGB1 was found to stimulate DNA binding of several steroid receptors including the* let-7* downstream target* AR* (Figure 10) [97]. These facts indicate that* HMGB1* may be a molecular marker for advanced prostate cancer [95, 96].

Although* HMGB1* was not shown to be a direct* let-7 *target, its expression is modulated by the direct* let-7* target HMGA1 [51]. Interestingly HMGB1 was also shown to be involved in the p53 network by facilitating the binding of the tumor suppressor p53 to its cognate DNA [98]. As mentioned before p53 can be inactivated by the HMGB1 sister protein HMGA1 by translocation of the p53 activator HIPK2 [51] (Figures 4 and 10). The tumor suppressor p53 in turn was found to downregulate the activity of the HMGB1 promoter [99] and to trigger the radiation induced decrease of* let-7a* and* let-7b* expression (Figures 4 and 10) in the human colon cancer cell line HCT116 [100].

## 6. *CCND2*

Many tumor cells accumulate mutations resulting in uncontrolled proliferation due to direct or indirect deregulations of the cyclin-dependent kinases (CDKs). Cyclins are known regulating subunits of CDKs being expressed at specific time points during the cell cycle. Consequently cyclin deregulations induce uncontrolled cell proliferation [101].

The cyclin D2 (CCND2) is one of the cell cycle regulating factors. This gene, which is highly conserved among mammals, has been associated with human prostate cancer [11], gastric cancer [102], colon cancer [103], and leukemia [104].

**Figure 5:**
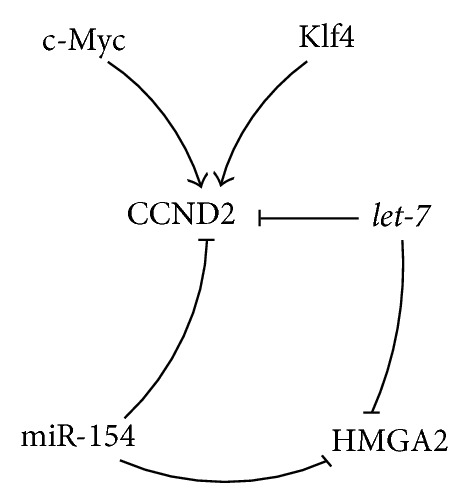
*Let-7* and CCND2 mediated gene regulation.

Interestingly,* CCND2* was shown to be a direct* let-7* and* miR-154* target like* HMGA2* [11, 41, 45, 105] (Figures 5 and 10). Additionally the* let-7* regulated oncogene* c-Myc* and the stem cell marker* Klf4* were reported to stimulate the* CCND2* transcription [106, 107] (Figures 5 and 10).

Dong et al. described that ectopically overexpressed* let-7a* induced cell cycle arrest at the G1/S phase by suppressing among others the cyclin CCND2 and additionally inhibited the proliferation of the human prostatic cell lines PC-3 and LnCap [11]. The same group reported that in nude xenograft mice, inoculated with* let-7a* transfected PC-3 cells, the tumor was 80% lighter after 4 weeks of growth compared to controls [11].

## 7. *c-Myc*

*c-Myc* is an oncogene frequently activated in human cancers, but is low expressed or absent in quiescent cells [108–110]. In contrast, its overexpression has been connected with PC formation and progression [111, 112]. This gene encodes a transcription factor that has a great impact on the global gene expression pattern and, thus, influences cell-cycle progression, glucose and glutamine metabolism, lipid synthesis, and many other processes, which contribute to tumor progression [109].

**Figure 6:**
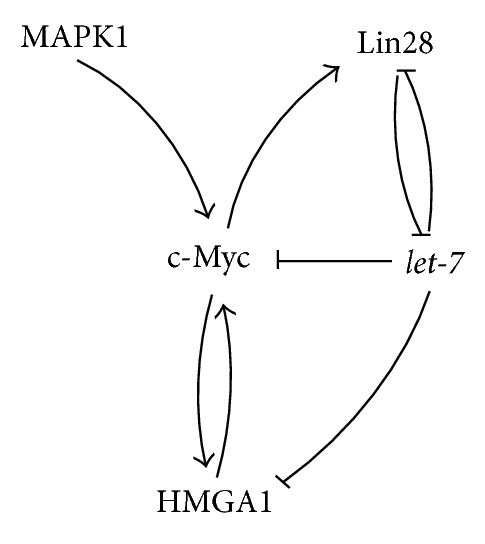
Interactions of the oncogene c-Myc with* let-7* and MAPK1, Lin28, and HMGA1.

Mitogen activated protein kinases (MAPK), glycogen synthase kinase 3 (GSK3), and other CDKs play a key role in the biological function and half-life of c-Myc proteins by posttranslational phosphorylation of the Thr58 end Ser62 sites [113] (Figures 6 and 10). Apart from various posttranslational protein modifications and transcriptional regulations of the* c-Myc* gene products, this gene was reported to be directly negatively regulated by members of the* let-7* family [114, 115] (Figures 6 and 10). Additionally, elevated MAPK1 activity, which was associated with advanced, androgen independent human PCs, [116] was demonstrated to influence the c-Myc protein, resulting in prolonged function in a human muscle-derived rhabdomyosarcoma cell line [117]. In line with the functions of c-Myc, MAPK1 controls diverse cellular processes as growth, differentiation, migration, and apoptosis, its deregulation has often been described to be associated with cancer [118]. Furthermore, c-Myc was shown to transcriptionally activate* Lin28* [119], which in turn inhibits the biogenesis of its regulator* let-7 *constituting a double negative feedback loop [47] (Figures 6 and 10). Interestingly the expression of the direct* let-7* target* HMGA1* is as well induced by c-Myc [48], which constitutes a positive feedback loop, stimulating* c-Myc* expression [50] (Figures 6 and 10).

## 8. *IL6*

Chronic inflammation of the prostate gained major attention as it is considered to account to the factors contributing to PC [120]. In previous reports a direct causal link between cancer and inflammation has been described with IL6,* let-7*, Lin28, and NF*κ*B being the major players involved in the epigenetic switch from inflammation to cell transformation [31].

Originally identified as an inducer of the terminal differentiation of B-cells into antibody-producing cells [121] interleukin-6 (IL6) appears to be a major regulator of prostate cancer progression [122]. Notably, IL6 is not only released by inflammatory cells but also found to be released by hormone insensitive cell lines DU145 and PC-3 but not by the hormone sensitive LNCaP cells [123]. Furthermore, this pleiotropic cytokine stimulates growth and survival of human PC and promotes its progression [123, 124]. In accordance, increased IL6 levels were found in epithelial cells of PC compared to benign tissues [125]. Moreover, Giri et al. reported a ~18 times higher IL6 expression in malignant prostate tissues compared to “normal” prostate specimens [126]. Michalaki and colleagues described significantly higher IL6 serum levels in patients with metastatic prostatic disease [127].

The biological activities of IL6 are mediated by binding to the *α*-subunit receptor IL6R and the following association with the ubiquitously expressed signal-transducing *β*-subunit gp130 [128]. Upon engagement of gp130 various Janus tyrosine kinase (JAK) family members (JAK1, JAK2, JAK3, and Tyk2) [129] are activated by ligand induced receptor oligomerization phosphorylating themselves and the intracellular domains of the receptors [130]. Once gp130 is phosphorylated the second protein family, the signal transducer and activator of transcription (STAT), binds to the intracellular domain of the receptor. This leads to the activation of STATs and the subsequent dissociation, allowing STAT dimerization and translocation into the nucleus where they act as transcription factors [131]. Additionally IL6 was shown to stimulate the PI3K and MAPK pathways by signaling trough activated gp130 [132, 133].

Interestingly, LnCaP cells stimulated with IL6 presented an enhanced AR activity in the absence of a ligand [134, 135]. The IL6 mediated activation of the human AR was indicated to be mediated by STAT3 and MAPK signaling [134, 136], which potentially contribute to recurrence of hormone refractory PCs. Whereas the AR transactivation can be suppressed by the PI3K/AKT pathway. Thus, these three pathways are suggested to coordinately regulate AR activation [136].

Acquiring resistance to apoptosis appears an important feature for the development of hormone resistant and aggressive human prostate cancer. Furthermore, IL6 was shown to act as a survival factor, blocking apoptosis induced by Bcl-xl, p53, TGF1*β* [137], and cytotoxic agents such as doxorubicin [138] and enzalutamide [139]. Whereas siRNA or STAT3-inhibitor-AG490 mediated suppression of the downstream acting STAT diminished the IL6 induced antiapoptotic function [138, 139].

**Figure 7:**
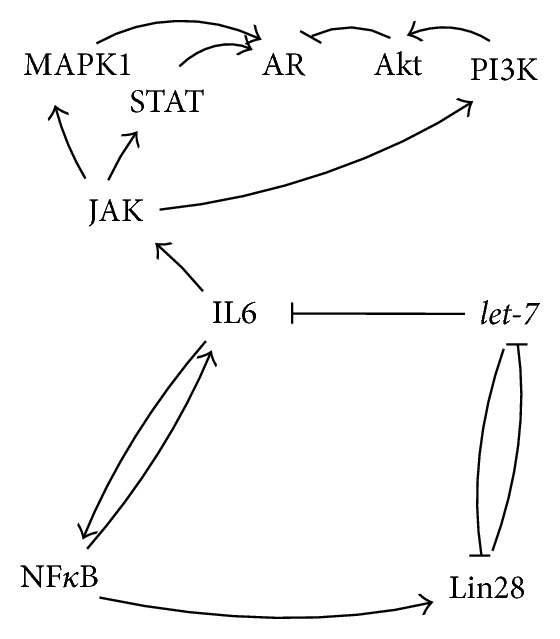
IL6 regulated genes.

NF*κ*B is a regulator of the transcription of* IL6* [140] and* Lin28B* [31, 141] (Figures 7 and 10). Lin28B was demonstrated to block the maturation of* let-7* [46]. Additionally, members of the miRNA* let-7* family directly target* IL6*, which in turn constitutes a positive feedback loop on NF*κ*B [31, 49] (Figures 7 and 10).

Remarkably, while only a few cells express membrane bound IL6R all cells display gp130 on their surface [132]. This is an interesting feature as IL6 can also bind to a soluble IL6R (sIL6R) variant, which interacts in an IL6R agonistic manner with gp130, thus, enabling the stimulation of cells lacking endogenous IL6R [142].

## 9. *RAS*

The founding members of the* RAS* gene superfamily* N-RAS*,* H-RAS,* and* K-RAS* are coding for small GTP-binding proteins [143]. Originally identified as retroviral oncogenes in rat sarcomas,* RAS* were the first human oncogenes discovered, shown to be mutated in around 30% of all human tumors [144, 145]. The very common mutations in the residues G12, G13, and Q61 lock RAS in a constitutively activated state by impairing the intrinsic GTP hydrolysis [145, 146]. RAS proteins are active when they have bound GTP. By hydrolyzing GTP to GDP they become inactive. The intrinsic GTPase activity of the RAS proteins is very low relying on the help of specialized GTP hydrolysis accelerating factors called GTPase activating proteins (GAP) which increase the hydrolysis by more than 100,000 fold [145].

**Figure 8:**
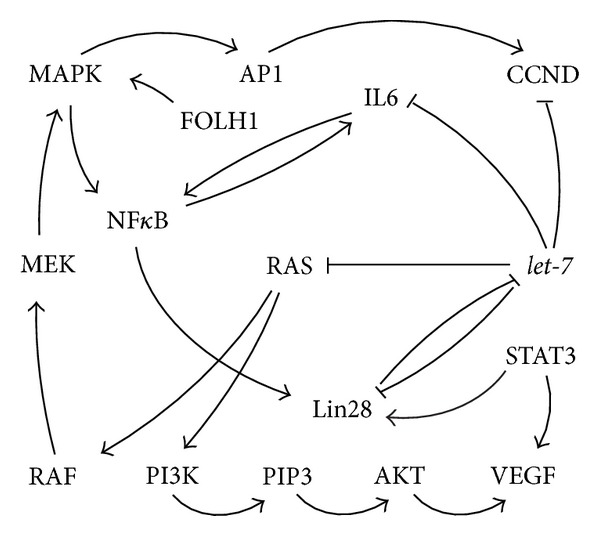
Schematic overview over some of the numerous pathways modified by RAS and* let-7*.

RAS-GTPs are acting as signal transducers across membranes by binding various effector proteins to stimulate signaling pathways [143, 147]. Among these factors are the Raf serine/threonine MAPKK kinases (ARAF, BRAF, and RAF1) which in turn activate MEK-MAPK cascades [148] (Figures 8 and 10). Accordingly, the mammalian MAPK pathways are estimated to be deregulated in one-third of all human cancers [149]. MAPKs activate cytosolic and nuclear factors like JUN and ELK1, which are regulating FOS expression. JUN and FOS are forming the activator protein 1 (AP1) and, thus, influencing the expression of proteins such as CCNDs which are involved in cell-cycle progression [150] (Figures 8 and 10).

Furthermore, RAS-GTPs induce the translocation and subsequent activation of phosphatidylinositol 3-kinase (PI3Ks) by binding to its catalytic subunit [151] (Figures 7 and 10). PI3K signaling is one of the most often deregulated systems in human cancer [152]. Taylor et al. described that the PI3K expression is altered in 42% of the primary and in 100% of the metastatic cases in the analyzed set of human prostatic cancers [153]. PI3Ks belong to one of the main effector molecules of RAS [151]. This enzyme type phosphorylates primarily to the 3′-OH group of the membrane bound phosphatidylinositol-4,5-biphosphate (PIP) to generate the messenger phosphatidylinositol-3,4,5-biphosphate (PIP3) [154]. PIP3 activates itself several pleckstrin homology domain-containing proteins as Akt by directly binding and recruiting it to the plasma membrane [154] (Figures 8 and 10). The activated Akt promotes many processes contributing to a malignant tumor phenotype [155]. Ectopic expression of a constitutively active Akt in the thyroid cell line SW579 was reported to significantly increase VEGF levels [156] (Figures 8 and 10). Neovascularization and angiogenesis are essential features for the progression of a growing tumor VEGF is one of the most important inducers of angiogenesis [157]. Niu et al. demonstrated a positive correlation between VEGF and a constitutively active STAT3 [157]. In accordance, it was found that STAT3 binds to the* VEGF* promoter [157] (Figures 8 and 10). Additionally STAT3 was reported to bind the promoter of the* let-7* biogenesis regulating gene* Lin28*, resulting in the concomitant upregulation of the* let-7* targets* RAS*,* c-Myc,* and* HMGA2* [158].

In human tissues the activation of RAS and Rac-MAPK pathways was described to be induced by the extracellular signal transducer FolH1 [159] (Figures 8 and 10). FolH1 is expressed in most of the human prostate cancers and is thus a potential target for diagnostic and therapeutic strategies [160]. The elicited phosphorylation of MAPK1 and MAPK14 induces in turn the activation of the transcription factor NF*κ*B (Figures 8 and 10) which controls the expression of various genes including the* let-7* biogenesis-controlling Lin28 [47] and the cytokine IL6 [31, 161] (Figures 8 and 10). Additionally, NF*κ*B was also shown to enhance the endogenous transcription of the primary miRNAs* let-7a-3 *and* let-7b* through NF*κ*B responsive binding sites in the promoter regions [141] (Figures 8 and 10).

Remarkably, Johnson et al. reported numerous* let-7* binding sites in the 3′-UTR of the RAS genes [42]. In conclusion the expression of the oncogenes* NRAS*,* KRAS,* and* HRAS* was described to be negatively regulated by several members of the* let-7* family [42, 162] (Figures 8 and 10).

## 10. *Androgen Receptor* (*AR*)

The gene of the steroid receptor family member* AR* [163] is located on the human chromosome X and codes for a ligand-dependent transcription factor [164, 165]. Upon ligand binding it translocates into the nucleus and regulates its target genes by binding to the androgen response elements (AREs) [166, 167]. Expressed in nearly all primary human PCs, AR plays a pivotal role in carcinogenesis of the prostate. At the initial diagnosis the majority of PCs depends on androgens and progress after hormone therapy to an androgen-independent disease [3, 168].

**Figure 9:**
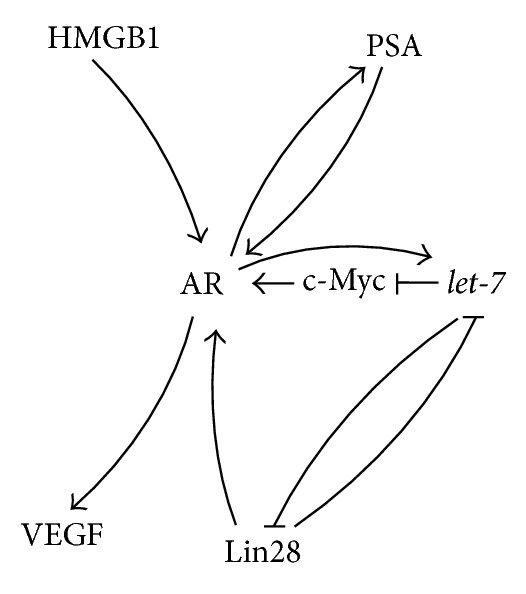
Potential AR interaction in prostate cancer development.

**Figure 10:**
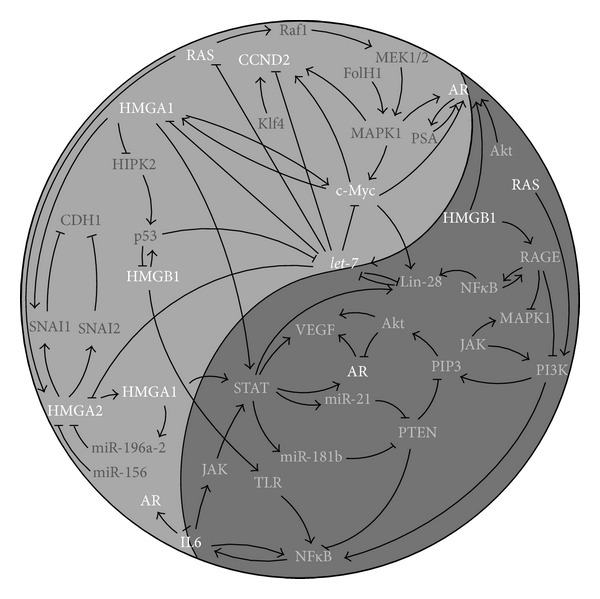
This figure represents the described interactions between* let-7 *and the reviewed* let-7* associated targets (in white letters) with other genes which are as well commonly deregulated in human cancers (in gray letters). The indicated interactions are on transcriptional, posttranscriptional or posttranslational level.

Continuous androgen expression is required to drive prostate gland formation during embryogenesis and later to maintain the normal function and glandular anatomy in adults [169]. In general the androgen mediated effects in prostate gland development are driven by the interaction with ARs [169]. The bypass mechanisms of* AR* upregulation include among others the HMGB1 enrichment on the* AR *promoter, which enhances the transcription [52] (Figures 9 and 10), an intracrine androgen production [170, 171] additionally ligand independent AR activation by cytokines or growth factors were reported as well [172]. Furthermore, altered specificity or sensitivity as for example by alternative splicing is discussed [173].

However, the activated AR stimulates the expression of its targets as, for example, the above mentioned VEGF [174] and PSA [175]. PSA is a pivotal downstream target of AR, which is used as biomarker for human PC progression [175]. Interestingly, the frequently observed rising of serum PSA in castrate-resistant PC patients could in parts be explained by AR activity, which is reexpressed/reactivated in advanced PCs [176]. Remarkably, PSA constitutes a positive feedback loop stimulating* AR* expression as was demonstrated* in vitro* [175] (Figures 9 and 10).

Furthermore, Tummala et al. highlighted the impact of the Lin28/*let-7*/Myc axis on PC and demonstrated that Lin28 activates the AR (Figures 9 and 10) and promotes growth of PC [177].

Remarkably* AR* was reported to be regulated in a negative way by the miRNA* let-7c *which suppresses its transcriptional activator c-Myc [12] (Figures 9 and 10). Additionally Lyu et al. described an AR induced upregulation of* let-7a, let-7b*,* let-7c*, and* let-7d* (Figures 9 and 10) in the breast cancer cell lines MDA-MB-231 and MDA-MB-453. At least in the case of* let-7a* this upregulation is indicated to be triggered by AR binding to AREs located at the* let-7a* promoter [53] (Figures 9 and 10). Furthermore, it was shown that in these cell lines the expression of the direct* let-7a *targets c-Myc and KRAS was decreased upon treatment with 5*α*-dihydrotestosterone and increased after an additional suppression of the miRNA* let-7a *[53].

The spatiotemporal expression of genes and functions depend highly on the cellular and developmental context. Thus, the impact of a single gene can be completely different between diverse tissues and at different time points in development. Nevertheless elucidation of the above described interactions in PC bears great potential due to the ubiquitous existence of the cellular regulatory elements and the potential interactions in each somatic cell of an organism. This idea is supported by the already found implication of each of the described genes in various human cancers. Furthermore several of the reviewed genes are already used as targets for diagnostic, prognostic, and therapeutic approaches. Thus, the master regulator family* let-7* is as well a promising target in cancer of the prostate gland.

For a better overview all described interactions between the master regulator family* let-7* and its major targets are summarized in Figure 10.

## 11. Conclusion

Although the knowledge of the genetic and epigenetic alterations in prostate cancer has significantly increased in the last decades, its diagnosis and therapy still remains a major challenge. The actually described genetic alterations in prostate cancer give more questions than answers. As we could highlight, the genes reviewed in the present paper are not acting in solitude but are closely interwoven with each other (Figure 10). Remarkably, the miRNA* let-7* family members are major players in the regulation of gene expression and appear to contribute greatly to the maintenance of the Ying and Yang in “normal” prostatic cells. However, their impact can be modified greatly by other factors. For that reason the complex intra- and intercellular genetic interactions of* let-7 *family members and associated genes must be further investigated and will likely have an impact on diagnostic, prognostic, and treatment modalities in future.
